# Remote methods for research on violence against women and children: lessons and challenges from research during the COVID-19 pandemic

**DOI:** 10.1136/bmjgh-2022-008460

**Published:** 2022-11-17

**Authors:** Amiya Bhatia, Ellen Turner, Aggrey Akim, Angel Mirembe, Janet Nakuti, Jenny Parkes, Simone Datzberger, Rehema Nagawa, Mary Kung'u, Hellen Babu, Rhoda Kabuti, Joshua Kimani, Tara S Beattie, Ana Flavia d'Oliveira, Poonam Rishal, Robert Nyakuwa, Sadie Bell, Paul Bukuluki, Beniamino Cislaghi, Clare Tanton, Anne Conolly, Catherine H Mercer, Janet Seeley, Loraine J Bacchus, Karen Devries

**Affiliations:** 1 Department of Global Health and Development, London School of Hygiene & Tropical Medicine, London, UK; 2 Raising Voices, Kampala, Uganda; 3 Institute of Education, University College London, London, UK; 4 MRC/UVRI and LSHTM Uganda Research Unit, Entebbe, Uganda; 5 Partners for Health and Development in Africa, Nairobi, Kenya; 6 Preventive Medicine Department, Faculty of Medicine, University of São Paulo, São Paulo, Brazil; 7 Department of Nursing and Midwifery, Kathmandu University School of Medical Sciences, Kavre, Nepal; 8 Q Partnership, Harare, Zimbabwe; 9 Department of Social Work and Social Administration, School of Social Sciences, Makerere University, Kampala, Uganda; 10 NatCen Social Research, London, UK; 11 Institute for Global Health, University College London, London, UK

**Keywords:** COVID-19, Public Health, Cohort study, Cross-sectional survey, Qualitative study

## Abstract

Collecting data to understand violence against women and children during and after the COVID-19 pandemic is essential to inform violence prevention and response efforts. Although researchers across fields have pivoted to remote rather than in-person data collection, remote research on violence against women, children and young people poses particular challenges. As a group of violence researchers, we reflect on our experiences across eight studies in six countries that we redesigned to include remote data collection methods. We found the following areas were crucial in fulfilling our commitments to participants, researchers, violence prevention and research ethics: (1) designing remote data collection in the context of strong research partnerships; (2) adapting data collection approaches; (3) developing additional safeguarding processes in the context of remote data collection during the pandemic; and (4) providing remote support for researchers. We discuss lessons learnt in each of these areas and across the research design and implementation process, and summarise key considerations for other researchers considering remote data collection on violence.

**Summary box**

- There is limited research on how to conduct remote data collection on violence against women and children and most research relies on in-person data collection.
- As violence researchers, we reflect on our experiences of redesigning eight studies in six countries to include remote data collection methods during the COVID-19 pandemic.
- Shifting to remote data collection in violence research required adapting to a range of moveable and unpredictable conditions specific to each context.
- To conduct remote data collection in the pandemic, the following areas were crucial in fulfilling our commitments to participants, researchers, violence prevention, and research ethics: (1) strong research partnerships; (2) adapted data collection approaches; (3) additional safeguarding processes; and (4) remote support for researchers.
- Committing resources to the additional steps required to protect participant and researcher safety while using remote methods in violence research is essential.
- Remote data collection to directly measure experiences of violence should only be conducted in specific circumstances, when it is possible to ensure safeguarding and either when participants are already engaged in the study, and/or in the context of strong and well-established research partnerships.
- This study offers lessons learned and recommendations for whether -- and how -- to design and conduct remote data collection on violence.

## Introduction

Violence against women and children has become both more prevalent and less reported during the COVID-19 pandemic.^1–6^ Although researchers across fields have pivoted to remote rather than in-person data collection,^7–9^ remote research on violence has posed particular challenges. UNICEF, the United Nations Population Fund (UNFPA), the Sexual Violence Research Initiative (SVRI) and others outlined concerns with remote data collection early in the pandemic.^2 3 10–13^ These include participant safety, under-reporting of violence as participants could fear being overheard, and limited safeguarding amid overburdened health services and poorly functioning violence response services. Instead, experts recommended using secondary or administrative data, collecting retrospective data when safe to do so, or using proxy and indirect measures for violence.^2 3 10–13^

Interviewing women, children and young people about experiences of violence is often more reliable than using administrative or service data. For example, a meta-analysis with data from over 9 million participants showed that the prevalence of child sexual abuse was 12.7% if self-reported and only 0.4% if reported by health professionals, teachers or child services.^14^ Analyses of the Violence Against Children Surveys in six countries showed that the self-reported prevalence of physical violence was at least 60% and of sexual violence at least 10%, while formal disclosure of physical and/or sexual violence ranged from 1% to 25%.^15^ Interviewing children and young people about violence is also central to a commitment to child participation, which emphasises the pivotal role of children in research that concerns their lives.^16–18^

Since the start of the pandemic, several studies have used remote data collection to interview women, young people and children directly about violence.^19^ These have included: phone interviews and web-based surveys with children about violence,^20–22^ phone interviews with women about injuries, safety and conflict in the home and community,^23^ and the use of list experiments, vignettes and indirect measures to ask about violence.^24^

Prior to the pandemic, violence research was rarely conducted using remote data collection. A rapid review of remote data collection on violence found only 14 studies, all from high-income countries, of which only two included children.^25^ There is a need for further research that examines if, and how, violence research can be ethically and effectively done remotely. As a group of violence researchers, we reflect on our experiences across eight studies, some ongoing and some complete, that included remote data collection methods. Please see online supplemental file 1 for our author reflexivity statement. We briefly summarise the studies, reflect on how we designed remote research in line with ethical principles and good practices for violence research developed by WHO, the Centers for Disease Control and Prevention (CDC), UNICEF, UNFPA, and other organisations,^26–30^ and share our lessons to inform the work of violence researchers who are considering using remote methods.

10.1136/bmjgh-2022-008460.supp1 Supplementary data

## Studies included and approaches to remote data collection

We draw on eight studies collecting data on violence against women (n=4) and children (n=4) in Brazil, Britain, Kenya, Nepal, Uganda and Zimbabwe during the COVID-19 pandemic. None of these studies included remote data collection in their original design. Table 1 summarises violence questions included and approaches to remote data collection, consent and safeguarding. Six out of eight studies were redesigned to use remote methods to interview school-age children, adolescents and young adults, and adult women about their experiences of violence. In four studies, healthcare providers and local stakeholders were interviewed about violence either in addition to, or instead of, interviewing women or children. We include examples from two longitudinal or cohort studies (the Context of Violence in Adolescence Cohort (CoVAC) study in Uganda 31 and the Maisha Fiti study in Kenya^32^); one cross-sectional nationally representative survey, established in the late 1980s and carried out approximately decennially (the British National Surveys of Sexual Attitudes and Lifestyles (Natsal) pilot of remote study^33^); three qualitative studies (the CoVAC qualitative study,^34^ the Bantwana programme in Uganda^35^ and the Child-friendly Catholic Schools Study-Zimbabwe (CCSS-Z)); and two mixed methods studies (the HERA - Healthcare Responding to Violence and Abuse study in Brazil and Nepal). Four studies were linked to violence prevention interventions at the community, school or health facility level.^36–38^ Phone interviews were the most frequently used method. Other remote methods included video interviews or online questionnaires. In all cases but one, ethics committees approved remote methods. In the HERA study, the national ethics committee in Nepal did not approve qualitative telephone interviews about violence due to safety concerns.

**Table 1:** Approaches to remote data collection

Study and location	Participants	Violence questions included	Types of remote data collection	Who collected data	Remote consent	Safeguarding in the context of remote data collection
Violence against children						
**1. CoVAC study, Uganda (Qualitative)** Context of Violence in Adolescence Cohort study, Uganda	Young people aged 16–19 years.	Effects of COVID-19 on daily lives, on relationships in families, with friends, on schooling/work and impacts in their communities; discussion about their coping strategies, sources of support and views on lockdown measures.	Phone interviews (n=34).	Trained interviewers with prior experience working with the study population and in the study site, individually assigned to each participant to build a relationship over time.	Participants had given prior consent for the study. Consent for interview sought over the phone and researchers filled in written consent forms.	Counsellors on staff for phone counselling. Predefined referral pathways. Links to local child protection systems.
**2. CoVAC study, Uganda (Quantitative)** Context of Violence in Adolescence Cohort study, Uganda	Young people aged 15–27 years.	Physical, emotional and sexual violence from a caregiver, intimate partner, at work; witnessing violence.	Phone interviews (n=2355).	Trained interviewers with prior experience working with the study population and in the study site.	Participants had given prior consent for the study. Consent for interview sought over the phone and researchers filled in written consent forms.	Several counsellors on staff for phone counselling and in-person counselling. Predefined referral pathways. Links to local child protection systems.
**3. Bantwana programme study, Uganda** Parenting & protecting children from violence during the COVID-19 pandemic, qualitative, Uganda	Caregivers belonging to positive parenting groups. Key stakeholders (including community development officers, case workers, teachers and local council representatives).	Impact of the pandemic on caregivers’ lives and parenting practices and on social norms that underlie violence; approaches being used to prevent and respond to violence against children before and during lockdown; whether being involved with the Western Uganda Bantwana programme helped them with parenting during lockdown.	Phone interviews (n=87).	Experienced interviewers who received refresher training on ethical conduct and safeguarding.	Consent for interview sought over the phone and recorded using digital recorders.	Predefined referral pathways. Links to local counselling and child protection services.
**4. CCSS-Z study, Zimbabwe** Child-friendly Catholic Schools Study, Zimbabwe	Adult Catholic school stakeholders (teachers, headteachers, parents, priests). External stakeholders (national Catholic education actors, NGO actors, local government education actors).	Violence against children in the community; teacher violence in schools, a child protection policy and intervention to prevent violence; and alternative discipline approaches in schools.	Face to face interviews (n=16), phone/video interviews (n=2).	Trained interviewers with prior experience conducting research into violence and conducting remote methods, but no prior experience with the study population.	Consent for phone/video interviews sought over the phone and recorded using digital recorders (consent was a three-stage process).	Predefined referral pathways with Childline Zimbabwe, with capacity for in-person referrals for children. Phone numbers offered for referral services for adults.

Violence against women						
**5. Maisha Fiti study, Kenya**	Female sex workers aged above 18 years.	Physical, sexual, emotional and economic violence ever and in the past 6 months from intimate partners, clients, police and others; measurement of gang rape, police arrest and police demands for sex to avoid arrest.	Phone interviews (n=26).	Trained interviewers with prior experience working with the study population and in the study site.	Participants had given prior consent for the study. Consent for remote interview sought over the phone and recorded using digital recorders.	Tele-counselling and telemedicine provided over the phone.
**6. Natsal study, Britain** British National Surveys of Sexual Attitudes and Lifestyles (pilot of remote study), Britain	Men and women aged 18–59 years in Britain.	Emotional, physical IPV in the past 12 months, ever experienced sexual violence.	Face-to-face (n=74), phone (n=47) and video (n=7) interviews all combined with an online self-completion questionnaire for more sensitive questions (which include questions on violence).	Trained interviewers with prior experience conducting face-to-face interviews for Natsal and/or other sensitive surveys.	Interviewers made contact with sampled participants in-person and agreed mode of interview. Participant preferred mode of interview was face to face; however, video or telephone interviews were offered to participants who did not wish to participate face to face. Verbal consent was obtained immediately before the start of the interview and recorded electronically by the interviewer.	Information leaflet provided (on paper and electronically) with websites and phone numbers for relevant services. A Disclosure of Harm Policy was in place.
**7. HERA study - Nepal** Healthcare Responding to Violence and Abuse, Nepal	Healthcare providers, in five health facilities in outreach centres of Dulikhel Hospital in province 3. Key stakeholders (eg, municipal health section, women’s referral centres), women who have experienced domestic violence.	Domestic violence against women by partners or any other family member.	Health providers: interviews with web-based questionnaire using KOBO toolkit (n=35), most significant story interviews (n=30). Survivors of violence: colour-coded audio computer assisted self-interview on tablets (n=45).	Trained interviewers and trained healthcare providers.	Consent was sought verbally and audio recorded. For healthcare providers, the consent form was attached to the online questionnaire.	For survivors, trained healthcare providers linked them to the focal person of related sectors via telephone (eg, focal person of one stop crisis management centres and lawyer via hotline). Researcher and participant safety and distress protocol.
**8. HERA study – Brazil** Healthcare Responding to Violence and Abuse, Brazil	Healthcare providers, healthcare managers in eight primary health clinics in the west and south regions. Key stakeholders (eg, municipal health departments, women’s referral centres) and women who have experienced domestic violence.	Domestic violence against women by partners or any other family member.	Interviews on Google Meet (55 healthcare providers, 12 managers and 5 survivors), online case discussions with a team of healthcare providers (38 meetings).	Trained female interviewers.	Consent form sent to healthcare providers before the interview and verbal consent was sought.	Referral list made and updated based on which services were operational in-person or remotely. Researcher and participant safety and distress protocol.

## Reflections on conducting remote data collection on violence during COVID-19

Most literature on the ethics of violence research describes the importance of the following principles: (1) strong partnerships and trained researchers who can build rapport with participants, sense distress and protect participants from harm, (2) privacy and safety, (3) strong links to local violence response services and referral organisations for safeguarding, and (4) training, support and debriefing for researchers.^26–29^ We discuss how we redesigned our research to fulfil each of these ethical principles, summarising key decisions and challenges. We offer case examples in table 2 and lessons learnt in table 3.

**Table 2:** Case studies in preparing for remote data collection

Principle	Case studies on conducting remote data collection on violence during COVID-19
(1) Designing remote data collection in the context of strong research partnerships	**Maisha Fiti study, Kenya** Research conducted with female sex workers in Kenya faced challenges, as many women lived with children, sometimes partners or other family members, and experienced insecure housing and work patterns due to the pandemic. At the same time, however, we also noted that working with sex workers could be different to other populations of women as they more often live without partners. We therefore felt that the risks were lower than with studies with cohabiting or married women, for example. Furthermore, we also noted some benefits of a flexible and remote approach with participants, as the study team was able to reach participants who would otherwise have been unable to participate due to migration and unstable housing, caused by economic difficulties of the pandemic. Here, we note how working with specific populations may offer certain challenges, but also particular opportunities for engagement. Remote methods research on violence highlights the impossibility of a one-size-fits-all approach and emphasises the need for participant-specific planning. **CCSS-Z study, Zimbabwe** In the CCSS-Z study, the research team did not feel the relationships with school staff and parents were in place to conduct remote interviews about violence. At the time of data collection in Zimbabwe, the government had recently signed into law Education Act Amendment No. 15 (2019) prohibiting corporal punishment in schools. This period of change had led to a sense of insecurity and sensitivity around this topic, and an increased media focus, with some undercover media reporting on violence using photos and video footage being published and shared on social media. This had heightened a generalised sense of distrust in and around schools of researchers, particularly for those making approaches over the phone, and a sensitive social and political context to be asking questions about violence in schools. Our researchers were highly cognisant of this and advised adapting the study design to avoid remote interviews with teachers and to place great emphasis on trusted pathways and partners through which we approached participants. Researchers waited until it was safe to conduct interviews with school staff and parents in person. Researcher knowledge of the context was essential, as well as a built-in capacity for the study to be adaptive and responsive to contextual needs of the moment. **Natsal study, Britain** The Natsal study is led by a multidisciplinary team of researchers across academic institutions and a national social research organisation. In addition, Natsal has a number of collaborators to support the topics addressed in the survey. Input into the questions and question wording for the sexual violence module in the fourth round of Natsal was obtained from researchers, practitioners and survivors’ organisations working in this field. The study was able to draw on the expertise and knowledge of survey methodologists to adapt the study design and data collection protocols for remote methods. The team considered and assessed a range of remote data collection models in terms of their ability to deliver the key design features of the Natsal study (i.e. probability sampling, minimising response bias, questionnaire length and sensitivity, a combination of interview and self-completion questions, biological sampling and maintaining a 30-year time-series). A recommendation was made to implement an interviewer-administered approach where initial contact was made in-person, face-to-face data collection offered with alternative remote modes available (video or telephone interviews with an online self-completion questionnaire). The team made significant adaptations to various aspects of planned study: fieldwork documents were modified, the interview instrument was adapted for remote modes, the self-completion was converted into an online questionnaire, remote biological sampling protocols were developed, paper consent forms (for biological samples and data linkage) were converted into eConsents, remote incentive administration was established and researcher training was adapted for online delivery.
(2) Safety and privacy of remote methods	**Bantwana programme study, Uganda** In our original research plan, in addition to interviewing caregivers and a range of adult stakeholders, we intended to conduct in-person focus groups with children in school settings. In response to the COVID-19 pandemic, we altered our research design and decided to conduct remote interviews with caregivers and adult stakeholders as we considered that remote data collection with children was not appropriate. This was primarily due to safeguarding concerns and practical constraints, such as how to safely reach children without going through schools. **Natsal study, Britain** In the Natsal survey, if a participant selected that they ‘prefer not to answer’ for any of the questions on violence, a question appeared asking whether they wanted to skip to the end of the section. Within the violence modules, there was also a button that the participant could click to take them to a neutral news website if there were issues with privacy. Throughout the online questionnaire, on completion of a section of questions, each section was locked to prevent the possibility of anyone going back to view previous answers. All participants taking part in remote method data collection were provided with an interview document pack containing participant facing materials (e.g. the participant information leaflet, interview show cards, a signposting leaflet). **Maisha Fiti study, Kenya** Prior to the main interview, the study team designed a process to have initial phone calls with participants to understand if they were interested in participating and to assess safety in their current location. Study procedures were also explained, and a time and day for the interview was agreed on. Technological challenges were also assessed and researchers discussed the following with participants: charging their phones, phone audibility, locating network friendly points, finding a safe place where people would not eavesdrop, and agreeing a safe word to terminate the interviews.
(3) Safeguarding processes in the context of remote data collection	**CoVAC study, Uganda** As part of the process of moving to remote methods, we engaged in a substantial revision to our directory of referral organisations. We contacted all referral organisations that we had previously worked with to find out if they were still operating during the pandemic period and if they could take up ourreferrals. As several had closed or we had lost contact with some of our contact persons, we added further organisations to our directory. Local organisations were prioritised to hasten the referral process in case of severe cases that required immediate in-person engagement. Given the effects of the lockdown on the economic situation of participants, we also sought local organisations that could provide social services or support with income generation. All local organisations were contacted by the study counsellor, and formal letters were sent as a follow-up to share information about the study. In the quantitative study—where we asked questions about violence—we offered tele-counselling to every participant. In survey programming, we embedded algorithms in the questionnaire to help to identify which participant needed what type of referral intervention. All participants that met any of the referral criteria and accepted counselling were assigned to study counsellors for phone counselling. In the qualitative study, telecounselling was offered to those participants who reported violence and were willing to talk to the counsellor. All telecounselling was done by full-time study counsellors and any other referrals that needed in-person engagement were coordinated by the study’s lead counsellor. **CCSS-Z study, Zimbabwe** In in-person interviews in a Catholic school, one headteacher disclosed knowledge of violence against a child in his school. This met the criteria for referral within our mechanisms; however, the headteacher assured the researcher that they were handling the case internally within the school’s existing referral mechanisms and requested that it was not referred to our partner organisation, Childline Zimbabwe. This left the researcher in a difficult position as she felt uncomfortable forcing a referral to our partner organisation and did not want to over-ride the headteacher’s practice within the school and threaten relationships. Furthermore, the school had robust and comprehensive child protection mechanisms in place due to a prior child protection programme being conducted. At the same time, she had concern for the child and the case warranted referral based on the referral protocol. The study team met with our referral partner organisation, Childline Zimbabwe to discuss this case. We determined a course of action to support the researcher and amended our referral protocol to reflect the particularities of this case, in case something similar arose in the future. While this case occurred during face-to-face research, the ethical quandaries it posed were heightened by the researcher connecting with the study team remotely, feeling more isolated than would otherwise be the case, in dealing with this tricky decision. This highlighted the importance of remote support for researchers conducting violence research remotely and highly detailed referral mechanisms.
(4) Remote support and training for researchers	**HERA study, Nepal and Brazil** The HERA study developed a researcher distress protocol that partners adapted to the local context. The protocol described the processes for responding to and referring women who experienced distress during interviews, or who disclose traumatic experiences for which they wish to access psychological support. The protocol provided examples of mild, moderate and major distress symptoms and potential responses. It made clear that the researchers should not act as a counsellor, but rather offer reassurance, empathetic listening and referrals to appropriate sources of support. In terms of researcher safety and well-being, the protocol outlined a number of steps to minimise risk when undertaking interviews (e.g. sharing details of field visits in advance, working in pairs, using a checking-in system). It also recommended debriefing and support for researchers (e.g. regular team debriefing meetings with the PI to discuss emotional aspects of the work and address any particular issues that have arisen, or to ensure access to psychological support if needed). In addition, study teams in Nepal and Brazil had daily debriefs and expanded researcher training to include training on remote data collection. Data packages were also purchased for all researchers. **CoVAC study, Uganda** In anticipation of an increase in COVID-19 cases, we amended our study protocol to include measures to facilitate remote training and data collection to ensure safety of both the researchers and participants. These measures included carrying out an assessment with researchers to understand the feasibility of home-based data collection, providing researchers with additional training on data management and storage, and purchasing opaque folders and headphones for all researchers. We also ensured all study tablets and mobile phones were password protected and encrypted, developed a code of conduct for home-based data collection, and conducted regular well-being debriefs with researchers. **Natsal study, Britain** The Natsal study included both in-person and remote data collection options. The study introduced COVID-19 fieldwork protocols and training to ensure the safety of participants and researchers during doorstep contact and face-to-face interviewing. The guidance included regular personal health checks for researchers, social distancing and hygiene measures, PPE provision, materials and equipment handling and participant COVID-19 screening. The researchers also attended video interview training that covered the protocols for setting up and administering interviews using Microsoft Teams.

**Table 3:** Lessons from remote data collection on violence

	Lessons from remote data collection on violence
Study (re)design	Assess whether it is safe, ethical and feasible to interview women, young people and children about violence using remote methods. Explore opportunities to conduct remote data collection with stakeholders and caregivers on violence against children. Limit remote data collection on personal experiences of violence to circumstances and in contexts where prior relationships have been developed with participants. Engage a study team with prior training and experience collecting data on violence. Build in additional staffing and support for researchers in order to offer participants flexibility with the days and times they can participate in interviews. Build on previously established relationships and rapport with participants to ensure trust and confidence in participating in remote interviews. Ensure timelines and budget allow for a redesign of the study to use remote methods. Budget lines should support funds for internet/data for researchers, and additional time to do interviews, as well as minimising mode effects when switching methods. For quantitative surveys, design questions with response options that are not sensitive, for example, ‘yes’, ‘no’, ‘many times’. For qualitative methods, avoid direct questions on personal experiences of violence so that the participants are able to maintain control over any personal disclosures.
Ethics	Draw on in-country guidance on data collection during COVID-19 and ensure all aspects of the study design are fully approved by local research ethics committees. Draw on guidance from WHO, the Centers for Disease Control and Prevention (CDC), UNICEF, the United Nations Population Fund (UNFPA), and other stakeholders on conducting remote data collection on violence. Write up, describe and be transparent about ethical considerations and challenges. Exercise ongoing ethical judgement – establish debriefing/check-in groups about learnings and quandaries as they emerge.
Training	Include training on remote data collection and data protection. Train researchers to listen for distress, discomfort, interruptions and how to handle silences. Acknowledge and be aware of power hierarchies that may prevent researchers from raising concerns. Offer training on researcher self-care with strategies for enhancing group connectivity when conducting remote violence research.
Data collection	Design data collection to respond to the availability of participants and plan study staffing accordingly. Build in safety checks and the process for interruptions. Develop protocols for how to respond if someone else takes the phone away, or safety is compromised. Have multiple platforms available for remote connection to participants. Use secure platforms for data collection and storage (eg, a study office where all data collection takes place, encrypted and secure tablets). Build in contingencies for COVID-19 related delays.
Safeguarding	Redesign referral and safeguarding processes for remote data collection. Engage counsellors to be part of the study team, or ensure referral lists for external organisations developed prior to COVID-19 are updated in the context of COVID-19. Ensure organisations in the referral network are functioning, phone numbers are updated, and organisations are accepting referrals before data collection begins. Offer telemedicine and telecounselling by experienced and trained staff, preferably known to participants. Check in to support researchers remotely with any sensitive emerging safeguarding issues.
Support to researchers collecting data	Provide a safe space and access to a counsellor. Include structured weekly debrief sessions with study Principal Investigators or the study coordinator to help troubleshoot any challenges and provide space for interviewers to discuss issues affecting them. Have regular check-ins and meetings to discuss wellbeing, assess challenges and make adaptations to better suppor researchers. Use WhatsApp groups with regular contact if helpful. Practise empathy for the context and situations of researchers.

### Drawing on strong research partnerships

Established relationships were essential for remote violence research: partnerships and teams had been in place for 1–7 years (and the infrastructure for Natsal had been in place for 30 years). Research partners had expertise in violence research, appraised the COVID-19 situation, sought approvals, and engaged trusted counsellors and referral networks for safeguarding. Strong research partnerships allowed remote data collection to be conducted by trained interviewers with prior experience of sensitive data collection on violence, building rapport with participants, and who, in many cases, were already engaged in the study.

The shift to remote methods was planned and discussed in the context of these pre-existing collaborations. We found that it was possible to ask directly about violence remotely in some cases but not in others. The six studies that did use remote methods to interview women, children and young people about their experiences of violence were ongoing studies or, in the case of Natsal, contact was made with participants prior to the interview. Moving to remote methods in these studies offered possibilities to increase, or enable, participation. For example, in the Maisha Fiti study, phone interviews reached female sex workers who would otherwise have been unable to participate due to COVID-19 response measures, as well as migration and unstable housing caused by economic difficulties of the pandemic (table 2). In Natsal, some participants mentioned in follow-up interviews that remote options could even offer more privacy to answer survey questions. In several studies, we felt continuing data collection was part of our ethical commitments to participants and related to improving violence services in a pandemic. In the CoVAC qualitative study, where researchers had been speaking to participants since 2018, halting contact with participants seemed unethical: in fact, young people appreciated that researchers followed up on their circumstances and reached out to them during the challenging time. In the HERA study, continuing data collection was seen as a source of hope and optimism for health providers and researchers, as study activities were central to improving health systems’ responses to violence. In the Natsal study, cognisant of the challenges of remote data collection, researchers designed a pilot before proceeding with large-scale remote data collection and paused the pilot for 12 months for the team to draw on the expertise of experienced survey methodologists to adapt the study design for remote delivery. When fieldwork began, COVID-19 restrictions allowed interviewers to make initial contact with participants on the doorstep and offer either a face-to-face or remote interview.

In contrast, in two new studies—the CCSS-Z study in Zimbabwe and the Bantwana programme study in Uganda—research teams were concerned about initiating data collection remotely without pre-existing relationships or contact with participants. Concerns also included identifying and safeguarding participants during school closures, building rapport with children remotely, and making virtual sessions engaging for children. These teams either delayed interviews with children, or decided not to interview children and instead interviewed adult stakeholders, such as teachers or parents, to gather some information about violence during the pandemic. It was deemed in both studies that these adults could offer critical insights into children’s experiences of violence during pandemic conditions that should not be missed, provided the studies could be adapted to meet ethical requirements. This required substantial changes to the study design, research approach and interview questions. Remotely recruiting adults to discuss violence was also challenging, however. In the Bantwana programme study, staff known to adult participants approached them initially before connecting them with the research team. In the CCSS-Z study, the research team did not feel the relationships with school staff and parents were in place to conduct remote interviews about violence, which was heightened by the political environment at the time (table 2). The study was further revised and remote interviews were only conducted with higher level stakeholders, external to the schools, who were accustomed to discussing violence in their work and to working remotely during the pandemic. In both these cases, the lack of prior relationships between the research team and adult participants meant that making initial contact through project staff or study partners who were known to and trusted by participants was a crucial first step. However, it is possible that participants may not have been as forthcoming as if they had had strong prior relationships with the research team. These experiences highlight the nuances of initiating violence research remotely, even when not asking about personal experiences of violence.

### Safety and privacy

To enable safety and privacy, study approaches were amended in four primary ways. First, consent and introductory processes were adapted for remote data collection to reduce risk of retaliatory violence and improve confidentiality. Violence was not mentioned while introducing the study: for example, Maisha Fiti means ‘life is good’ in Swahili, and the CCSS-Z study team initially referred to the study as a ‘Catholic Schools Study’ on the phone, only explaining further verbally when certain of speaking to intended participants. For most studies, consent was adapted to be sought verbally and documented either through audio-recording or by interviewers’ paper or electronic records. For Natsal, consent was sought in person at the doorstep of households where participants were offered a choice between in-person or remote interviews.

Second, we redesigned our interview scheduling. Across the studies, participants faced a range of challenges, such as additional time pressures, workloads, increased caregiving responsibilities or unpredictable working patterns. This meant that completing interviews in one sitting and keeping interviews confidential and private could be challenging. We found that data collection had to be highly adaptable and responsive. The Bantwana study halved the interview length to ensure phone interviews were manageable for participants. For both CoVAC studies and Maisha Fiti, we included an initial call to assess safety, explain study procedures and asked participants to suggest preferences for interview timing. We then designed processes for callbacks and offered flexibility around time so interviews could fit around the daily lives of participants. Planning ahead for staffing around this was necessary.

The reliability of phone or internet connection was also an ongoing challenge. This was a key concern for violence research, due to the risk of sudden disruptions to sensitive conversations, or being unable to reach participants in the case of safety concerns. In several cases we reimbursed participants to cover data used in communication, or researchers called participants, to both prevent participants from incurring costs and ensure sufficient phone credit for the call. In CCSS-Z, we also found that having more than one line of communication (establishing both online and phone contact) was also important in case of network failure. In Natsal, participants were emailed a unique URL to the online questionnaire during the telephone or video interview. At times this was slow to arrive, so the interviewer read the URL aloud. Video and phone were used to build rapport and interviewers remained on the telephone/video call while participants self-completed online questionnaires.

Third, interview processes were adapted to ensure privacy and confidentiality remotely. In line with good practices for research on sensitive topics, quantitative questions were designed with response options (e.g. ‘yes’, ‘no’, ‘few’) so it was not obvious participants were discussing violence. In the CoVAC qualitative study, topic guides were revised to ask participants open-ended questions, for example about how COVID-19 had affected their families, instead of direct questions about violence; and researchers took extra care around probing, for example on relationships or discipline at home. This approach enabled participants to maintain control over what they shared and allowed researchers to follow-up on sensitive topics when in-person interviews were possible. In the CoVAC quantitative study, the phone survey was redesigned to consist of three interviews, with more sensitive questions in later interviews.

Finally, researchers were trained to work with the participant to find a private place, to check if participants were alone, to change the subject, to listen out for signs of distress or discomfort, and to answer questions from other household members if they were to take the phone. In the HERA study, researchers would change the subject to reproductive health if the interview was interrupted. In the Natsal survey, an online self-completion questionnaire was used, with a button taking the participant to a neutral news website if privacy was lost. If a participant selected ‘prefer not to answer’ at any violence questions, an option appeared to skip to the end of the section. On completion, each section of questions was locked to prevent anyone viewing previous answers. In the CoVAC survey, ‘interruption’ options were added so researchers could log where they paused and call back later. In Maisha Fiti and the CoVAC survey, ‘safe words’ or ‘safe phrases’ were agreed with participants, so use of a phrase, such as ‘the weather is sunny’, or the name of a participant’s favourite football team or primary school, indicated that they were unable to continue the interview.

### Safeguarding processes

A further key consideration was how to maintain safeguarding and strong links to violence response services during the pandemic. Collecting data in person allowed counsellors to accompany participants to health or social services and provide in-person counselling. Collecting data remotely meant we were concerned about: (1) the functioning of health and social services or their ability to receive and support participants and (2) referring and accompanying participants. Study teams worked closely with referral partner organisations and adapted referral protocols in light of these challenges. The Maisha Fiti study adapted referral mechanisms to include telemedicine and telecounselling for participants who needed follow-up care for experiences of violence or mental health support: this was delivered remotely by the study counsellor who the participants had already met in person. The CoVAC study offered all young people phone counselling and referrals, and employed three experienced full-time counselling staff. In preparation, team members called over 50 local organisations to assess their functioning during the pandemic to create a new referral list of organisations. This helped in identifying focal point persons and establishing relationships before participants were referred. Similarly, in Brazil, the HERA team made a new referral list based on the availability of remote or in-person services. Natsal employed an organisation-level Disclosure of Harm Policy: researchers were trained to report incidents where they sensed risk of harm and a Disclosure Board (consisting of senior staff) made further decisions about intervention and further disclosure of participant information.

### Support for researchers

Finally, modifications were needed to design safe working environments and support for researchers conducting remote data collection. In addition to the inherent challenges of conducting violence research, all research teams experienced the stress and uncertainty of COVID-19 restrictions, and many researchers also experienced bereavements or tested positive for COVID-19. To protect and respond to researcher well-being, the HERA study developed a researcher distress protocol, and the CoVAC study engaged an external counsellor to provide psychosocial support to researchers. Other strategies used across the studies included: locally produced guidance for prioritising safety, daily debriefs, zoom polls and WhatsApp groups for regular contact and motivation. Some studies provided time off for vaccinations, additional payments to offset some of the negative economic consequences of the pandemic, and developed a daily schedule to fit with caring and childcare responsibilities. For example, the CoVAC study provided a food allowance during remote researcher training so researchers could support their households.

We also created work environments where researchers could make confidential phone calls. The Maisha Fiti study used a study office from where researchers could make calls, with COVID-19 safety measures. In several cases, it was not possible for researchers to work from an office or health facility. Following a lockdown in Uganda, the CoVAC study shifted to home-based data collection and designed additional measures to support researchers and protect data. Study staff visited each researcher at home to discuss home-based data collection, privacy considerations, strategies for data storage and internet connectivity. Lessons from these conversations informed a code of conduct for home-based research, and the purchase of power banks, mobile phones, comfortable headphones and opaque folders for researchers, which were delivered to their homes.

## Our learnings from remote data collection and ways forward

Table 3 summarises our key learnings from redesigning violence research during the COVID-19 pandemic. These lessons may be helpful beyond COVID-19 and have implications for researching other sensitive topics more broadly, and for research on topics where participants may disclose violence. We found that shifting to remote methods to conduct violence research requires adapting to a range of moveable and unpredictable conditions specific to: the social and political context of each study, the local COVID-19 response and the study design, and the partnership arrangements in place. Committing time and budget to the additional steps required to protect participant and researcher safety^39^ is essential. These lessons both affirm, and build on, much of the existing guidance.^2 3 10–13 25^ Namely, that remote data collection to directly measure experiences of violence should be conducted in specific circumstances, when it is possible to ensure safeguarding and either when participants are already engaged in the study, and/or in the context of strong and well-established research partnerships. In some cases, remote methods allowed us to fulfil our commitments to participants and maintain relationships during the pandemic, and in other cases, remote research prevented us from doing so. When it was not possible or safe to directly interview women and children about violence using remote methods, we used other approaches to engage participants and generate evidence on violence, acknowledging the limitations of not interviewing women and children directly. Although remote research should not replace face-to-face research on violence, these approaches could be combined. We found some participants appreciated the convenience of remote interviews, finding it easier to fit in participation alongside other commitments.

There remains much to learn about the ethics, possibilities and limitations of remote data collection methods. In this paper, we offer insights into how research teams selected and used a range of remote methods, and discuss how these approaches were developed and implemented. We are unable to evaluate which remote method was most effective. There is limited evidence on whether remote methods or face-to-face interviews improve disclosure of violence in research, however evidence suggests that more anonymous data collection methods (e.g. a computer-assisted self-administered interview) could increase disclosure of violence.^40^ Future research could develop, compare and test approaches to remote data collection in different settings. Further research could explore how study participants experience remote data collection methods, assess the effects of remote methods compared with face-to-face methods on violence reporting, and examine how data collection through remote methods could be used to inform violence prevention and response. Our findings suggest that remote methods may be a way to reach marginalised groups in some contexts, and further research should also explore whether remote methods could be used to reach migrant populations and street connected young people, paying attention to safety, ethics, privacy, power dynamics and access to phones and the internet. It is important that future research meaningfully engages women, children and young people as co-creators of research on remote methods. Such work could provide improved guidance to researchers and to institutional and national research ethics committees who are also exploring the safety and ethics of these methods.

As Parkes and colleagues note, ‘the researcher often benefits more from the telling than the researched,’^34 41^ raising important questions that we, as researchers, should be asking ourselves as we strive to fulfil our commitments to research ethics, to survivors of violence, and to our research teams as we continue to generate evidence about violence against women and children to inform policy, practice and prevention.

## Data Availability

Data sharing not applicable as no datasets generated and/or analysed for this study.
