# Supplementary material for: Remote methods for research on violence against women and children: lessons and challenges from research during the COVID-19 pandemic
Source: BMJ Glob Health. 2022 Nov 17;7(11):e008460. doi: 10.1136/bmjgh-2022-008460 (PMC9676415; doi:10.1136/bmjgh-2022-008460)
Supplement: Supplementary data [file bmjgh-2022-008460supp001.pdf]

Remote methods for research on violence against women and children: lessons and challenges from research during the COVID-19 pandemic

Supplementary Material

Author Reflexivity Statement

1. How does this study address local research and policy priorities?

This study was motivated by the challenges local research teams faced in eight different studies across six different country contexts while re-designing and conducting research on violence against women and children during the COVID-19 pandemic. To ensure the research was safe and ethical we drew on the involvement, experience and expertise of the study teams, including both local and international partners. Together the team members could identify and address key issues highlighted in each setting. This study therefore aims to address the challenges of conducting ethical research on violence during and after pandemic conditions, paying close attention to the unique concerns in particular study contexts.

2. How were local researchers involved in study design?

In each of the eight studies included in this paper, local researchers are co-PIs or co-Is of the original study, and co-authors of this paper. This paper emerged directly from conversations research team members had during the pandemic, many of which were led by local researchers. To develop this paper, each research team – made up of local researchers and researchers in high income countries – shared content and examples for the paper and the first two authors led the writing process.

3. How has funding been used to support the local research team?

Each of the eight studies included are externally funded and involve strong partnerships with local research teams, which we describe in the paper. In most cases, these partnerships include funds for capacity building and co-learning which are linked to individual grants.

4. How are research staff who conducted data collection acknowledged?

Research staff across all the research teams are included as co-authors if they were substantially involved in data collection, analysis or writing for this paper. All study teams were offered the opportunity to participate in the writing of the paper. Other key research staff who contributed to data collection and study management but did not participate in developing this particular study have been listed in the acknowledgements.

5. Do all members of the research partnership have access to study data?

N/A – this is a practice paper and not based on study data.

6. How was data used to develop analytical skills within the partnership?

N/A – this is a practice paper and not based on study data.

7. How have research partners collaborated in interpreting study data?

N/A – this paper is not based on study data.

8. How were research partners supported to develop writing skills?

This paper engaged researchers from lower and middle income countries (LMICs) from all studies that were included in writing and/or revisions. All research partners were offered the chance to contribute written pieces for the study, where co-authors provided feedback.

9. How will research products be shared to address local needs?

This paper will be published open access. Our team of 25 co-authors who are based across 6 country settings and connected to local and global violence researchers and policy, will share this paper with local and global researchers and stakeholders. Through the process of collaboration and developing this paper, the study teams learned about remote methods and approaches used in other settings, and had the opportunity to critically reflect on learnings through our own study processes. This has strengthened both local and international study teams' capacity, and as many of the studies are ongoing this learning will strengthen ongoing practices in each setting.

10. How is the leadership, contribution and ownership of this work by LMIC researchers recognised within the authorship?

Seven out of eight of the studies we include are in five LMIC settings. LMIC researchers are co-Is or co-PIs of each of the studies and co-authors of this paper.

11. How have early career researchers across the partnership been included within the authorship team?

Early career researchers (AB, ET, AA) wrote and conceptualised this paper and are the first three authors.

12. How has gender balance been addressed within the authorship?

20 authors self-identify as female and 5 authors as male. Study teams were all mixed gender and were offered the opportunity to participate in the authorship.

13. How has the project contributed to training of LMIC researchers?

Most of the studies included have in-built capacity building and training in their study designs. As part of this paper, all study teams (including LMIC reseearchers) were offered the opportunity to take part in the writing of the paper. Indirectly, as mentioned in question no.9, the process of collaborating for this paper has also strengthened the capacity and critical reflection of all researchers on the use of remote methods.

14. How has the project contributed to improvements in local infrastructure?

This project has not directly contributed to improvements in local infrastructure.

15. What safeguarding procedures were used to protect local study participants and researchers?

We describe safeguarding procedures in detail in the manuscript as this is one the key aims of the paper.
